# USP22 drives colorectal cancer invasion and metastasis via epithelial-mesenchymal transition by activating AP4

**DOI:** 10.18632/oncotarget.15950

**Published:** 2017-03-06

**Authors:** Yongmin Li, Yanmei Yang, Jingwen Li, He Liu, Fuxun Chen, Bingyang Li, Binbin Cui, Yanlong Liu

**Affiliations:** ^1^ Department of Colorectal Surgery, The Affiliated Tumor Hospital of Harbin Medical University, Harbin 150081, China; ^2^ Center for Endemic Disease Control, Chinese Center for Disease Control and Prevention, Harbin Medical University, Harbin 150081, China

**Keywords:** USP22, liver metastasis, colorectal cancer, EMT, AP4

## Abstract

Ubiquitin specific peptidase 22 (USP22), a putative cancer stem cell marker, is overexpressed in liver metastases of colorectal cancer (CRC). However, the mechanism by which USP22 promotes CRC metastasis remains largely unknown. Here, we report that USP22 and AP4 are simultaneously overexpressed during TGF-β1-induced CRC cell epithelial-mesenchymal transition (EMT). USP22 up-regulation enhances CRC cell migration and invasion and EMT-related marker and AP4 expression, but these effects are partly blocked by AP4 knockdown. In addition, USP22 binds to the promoter region of AP4 to activate its transcription. *In vivo*, elevated USP22 expression promotes CRC cell metastasis to the lungs in nude mice, as evidenced by the fact that CRC metastatic nodules stain deeply positive for USP22 and AP4. In human CRC tissues, the genes encoding USP22 and AP4 are overexpressed in metastatic liver lesions compared with primary cancer tissues, and their overexpression is significantly associated with poor CRC patient survival. These findings indicate that USP22 and AP4 may serve as prognostic markers for predicting the risk of developing distant metastases in CRC.

## INTRODUCTION

Colorectal cancer (CRC), one of the most common malignant tumors, is the second most common cause of cancer mortality worldwide due in part to its high tendency to recur and metastasize [1]. There are no effective treatments that can selectively control CRC metastasis, and elucidating the molecular mechanisms underlying CRC metastasis and identifying effective disease prevention and treatment modalities remain important goals in CRC research.

Ubiquitin specific peptidase 22 (USP22), a deubiquitinating enzyme (DUB), is a member of a family of 11 gene signature-encoded proteins involved in the cancer stem cell (CSC) phenotypes of aggressive growth, metastasis and therapy resistance [2, 3]. Increased USP22 expression is associated with poor outcomes in multiple cancers [4–6]. We previously demonstrated that USP22 overexpression may contribute to CRC patient deaths and may play an essential role in distant metastasis [7]. USP22 can promote lung cancer cell invasion via epithelial-mesenchymal transition (EMT), which participates in the metastasis of primary tumors by activating TGF-β1 [8].

Among the multiple mechanisms underlying cancer cell metastasis, EMT has become a research focus in recent years. EMT is a complex molecular process involving changes in cellular phenotypes that entail the loss of epithelial markers, such as E-cadherin, and the gain of mesenchymal markers, such as N-cadherin and vimentin [9]. Transformation from the expression of E-cadherin to N-cadherin, a key marker of EMT, results in increases in cell migration, invasion and metastasis abilities [10, 11]. In many types of cancer cells, including bladder, liver, ovarian, breast and CRC cells [12], EMT reduces adhesion with basal cells to enhance invasion and migration ability, thus promoting tumor metastasis to distant organs [13–15]. Some transcription factors, such as TGF-β1, SNAIL, SLUG, ZEB1, ZEB2 and TWIST, can induce EMT [16, 17]. Activation of activator protein 4 (AP4), a basic helix-loop-helix and leucine-zipper transcription factor, was recently shown to induce EMT and enhance CRC cell migration and invasion. Elevated AP4 expression in primary CRC is significantly associated with liver metastasis and poor survival [18, 19]. And, AP4 transcription is increased by TGF-β receptor signaling activation [20], which is driven by USP22 [8]. In addition, the oncogene cellular myelocytomatosis oncogene (c-MYC) directly increases AP4 expression to trigger a transcriptional cascade [21], and USP22 is required for c-MYC-mediated transcription [22]. These findings suggest that AP4 may be involved in USP22-driven CRC progression and metastasis.

Here, we propose a novel mechanism by which USP22 drives CRC progression and metastasis. We demonstrate that USP22 and AP4 are simultaneously overexpressed in primary carcinoma compared with their non-cancerous mucosa and are both up-regulated in EMT in CRC cells. Moreover, USP22 up-regulation induces EMT by directly increasing AP4 transcription, resulting in CRC cell metastasis to the lungs *in vivo*. In addition, high levels of USP22 and AP4 are significantly correlated with liver metastasis and poor prognoses in CRC patients. These findings indicate that USP22 promotes CRC invasion and metastasis by inducing EMT via AP4 activation.

## RESULTS

### Elevated USP22 and AP4 expression in cancerous tissues and CRC cell EMT

**Figure 1:**
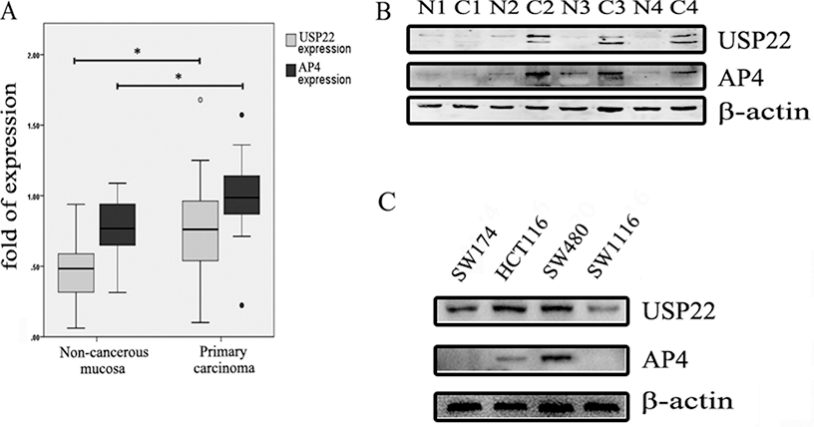
USP22 and AP4 expression in CRC tissues and cells (**A**) Western blot analysis of USP22 and AP4 levels in 19 pairs of non-cancerous mucosa and primary carcinoma. Both USP22 and AP4 were overexpressed in primary carcinoma relative to their expression in matched non-cancerous mucosa. (**B**) Four pairs of samples are shown as representative examples. (**C**) USP22 and AP4 protein levels were assessed in the CRC cell lines, containing SW174, HCT116, SW480 and SW1116, by western blot (WB). Significance level as indicated: *p < 0.05.

We performed western blot (WB) analysis of USP22 and AP4 expression in 19 primary CRC tissue samples and matched non-cancerous tissue samples. USP22 (*p* = 0.024, Figure 1A) and AP4 (*p* = 0.023, Figure 1A) expression levels were significantly higher in the primary CRC samples than in the matched non-cancerous mucosal samples. Representative results are shown in Figure 1B. USP22 and AP4 expression levels in four CRC cell lines, SW174, HCT116, SW480 and SW1116, were also assessed by WB. Both USP22 and AP4 expression levels were lowest in SW1116 cells and highest in SW480 cells (Figure 1C). In addition, transwell assays demonstrated that SW480 cell invasion ability was stronger than that of SW1116 cells (Supplementary Figure 1).

**Figure 2:**
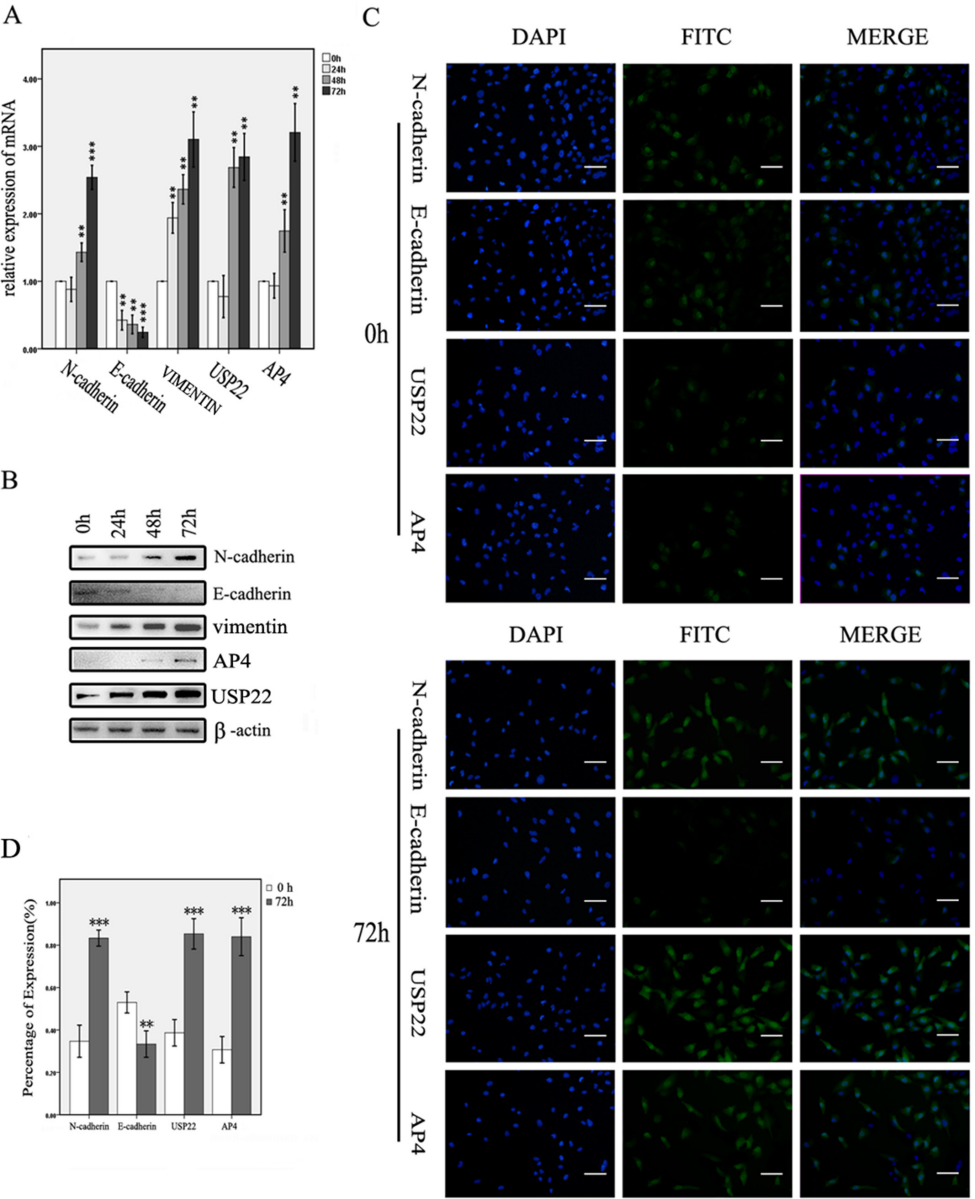
USP22 and AP4 expression in TGF-β1-induced EMT cells (**A**, **B**) RT-qPCR and western blot analysis of USP22 and AP4 expression in SW1116 cells induced with TGF-β1 for 0 h, 24 h, 48 h and 72 h. Gradually increases in the expression of N-cadherin, vimentin, USP22 and AP4 and decreases in the E-cadherin expression were observed at both the genetic and protein levels. (**C**, **D**) Immunofluorescence analysis of the expression of N-cadherin, E-cadherin, USP22 and AP4 in SW1116 cells induced by TGF-β1 for 0 h and 72 h. The percentages of N-cadherin-, USP22- and AP4-expressing cells were significantly higher, whereas E-cadherin expression was lower, in the cells induced for 72 h than in the control. Bars, 50 μm. Significance level as indicated: *p < 0.05; **p < 0.01; ***p < 0.001.

TGF-β1 was used to induce EMT in SW1116 cells. We selected the induction dose and time points based on the results of previous studies [23–25]. Changes in the expression of key molecules involved in EMT were detected by reverse transcription and real-time polymerase chain reaction (RT-PCR) and WB analysis. When the cells were treated with TGF-β1 from 0 h to 72 h, N-cadherin and vimentin expression levels gradually increased, whereas E-cadherin expression levels decreased, changes consistent with those that typically occur during EMT. USP22 and AP4 levels increased concurrently and gradually during this process (Figure 2A–2B). Similar results were obtained by immunofluorescence assay (Figure 2C–2D). These data suggest that USP22 and AP4 may be concurrently involved in CRC malignancy progression and metastasis.

### USP22 increases CRC cell migration and invasion by inducing EMT

**Figure 3:**
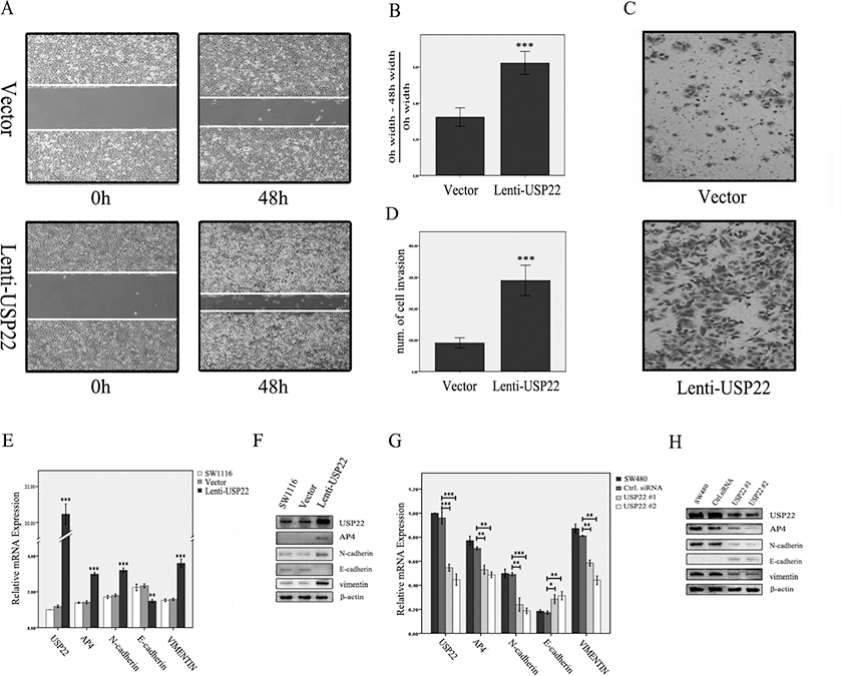
Effects of USP22 up- and down-regulation on CRC cells (**A**–**D**). Scratch and transwell assays showed that migration and invasion abilities were significantly increased in USP22-up-regulated cells compared with the control. (**E**, **F**). Up-regulation of USP22 resulted in increased levels of N-cadherin, vimentin and AP4 and decreased E-cadherin expression as assessed by RT-PCR and western blot (WB) analysis. (**G**, **H**) RT-PCR and WB analysis demonstrated that N-cadherin, vimentin and AP4 expression were decreased and E-cadherin expression was increased in SW480 cells in which USP22 was down-regulated. Significance level as indicated: *p < 0.05; **p < 0.01; ***p < 0.001.

To explore the functional role of USP22 in CRC, we overexpressed USP22 and detected the changes in cellular dynamics and expression levels. Scratch and transwell assays revealed that migration and invasion were significantly increased in USP22-up-regulated cells compared to migration and invasion in control cells (Figure 3A–3D). We then examined the changes in the expression levels of EMT markers. N-cadherin and vimentin expression levels were increased, whereas E-cadherin expression levels were decreased (Figure 3E–3F). To confirm these findings, we knocked down USP22 in SW480 cells and detected EMT-related factor expression levels. Consistent with the above observations, USP22 depletion decreased N-cadherin and vimentin expression levels and increased E-cadherin expression levels (Figure 3G–3H). These results indicate that USP22 increases CRC cell migration and invasion abilities by promoting EMT.

### USP22 induces EMT by activating AP4 transcription

**Figure 4:**
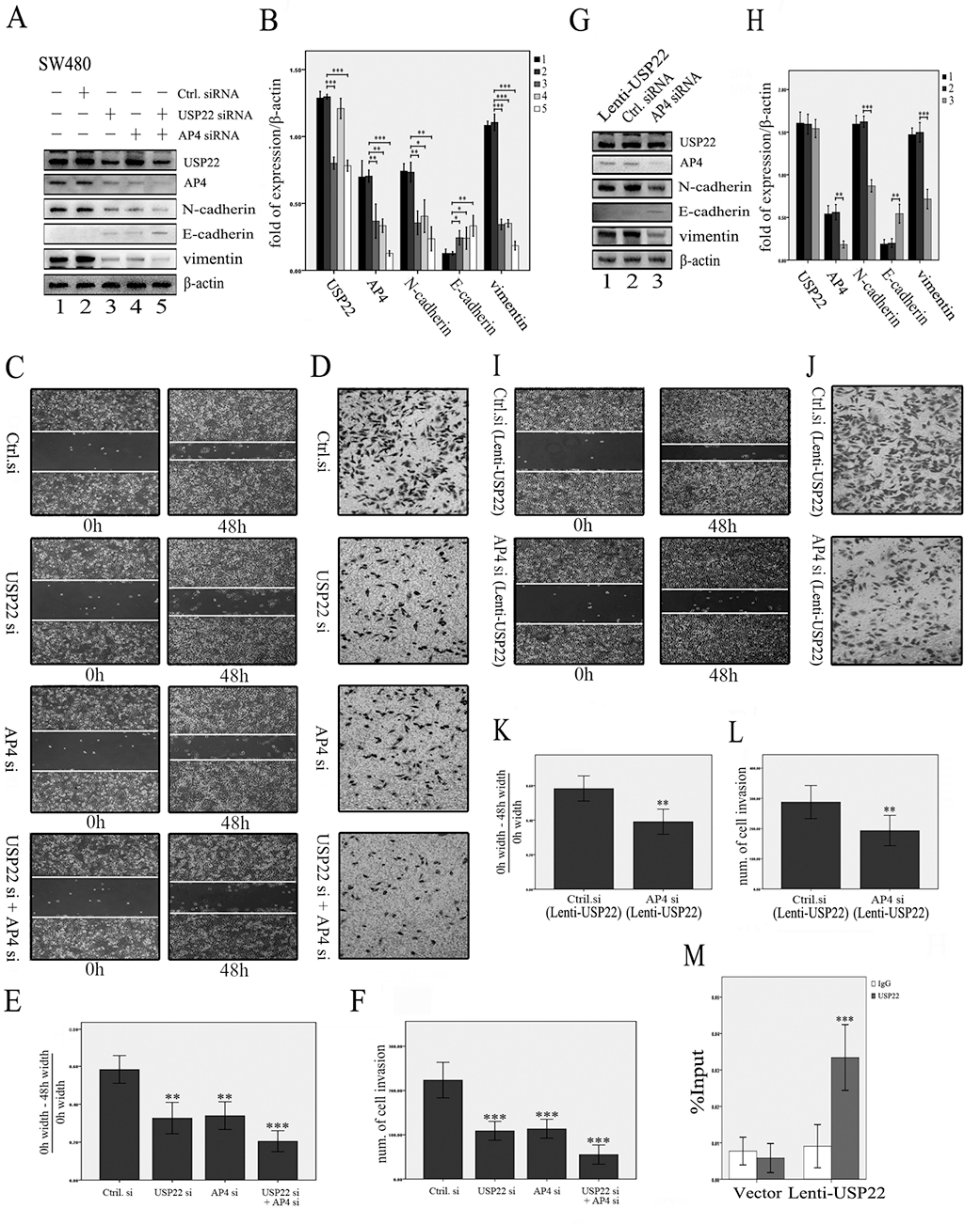
USP22 induces EMT by regulating AP4 (**A**, **B**) The effects of knocking down USP22 and AP4 in SW480 cells. Western blot (WB) analysis revealed that knockdown of AP4 resulted in decreased N-cadherin, vimentin, and AP4 expression and increased E-cadherin expression, similar to the effects of USP22 down-regulation, whereas USP22 expression remained unchanged in AP4-down-regulated cells. The extent of mesenchymal-epithelial transition was greater in the double-gene (USP22 and AP4) knockdown cells than in the other cell types. (**C**–**F)**. Similar results were observed in the scratch and transwell assays. (**G**, **H**) By contrast, WB analysis revealed that knockdown of AP4 reduced the expression of N-cadherin, vimentin, and AP4 and increased E-cadherin expression in lenti-USP22 cells (USP22 up-regulated cells), whereas USP22 expression remained unchanged. (**I**–**L**) Scratch and transwell assays revealed that the increased migration and invasion abilities resulting from USP22 up-regulation were significantly decreased in AP4 knockdown cells compared with the control. (**M**) ChIP analysis revealed that USP22 bound to the promoter region of AP4 (IgG served as a negative control). Significance level as indicated: *p < 0.05; **p < 0.01; ***p < 0.001.

As shown in Figure 3, USP22 activates AP4 expression during EMT induction. To explore the role of AP4 in the promotion of EMT by USP22, we knocked down AP4 in USP22-down-regulated cells to detect the changes in the expression levels of key factors involved in EMT. First, we determined that AP4, N-cadherin, E-cadherin and vimentin expression levels were similar in AP4-down-regulated cells and USP22-down-regulated cells, findings consistent with those of a typical mesenchymal-epithelial transition, but USP22 expression remained unchanged in AP4-knockdown cells. The extent of mesenchymal-epithelial transition in double-gene (USP22 and AP4)-knockdown cells was greater than that in other cells (Figure 4A–4B). Similar changes in cell migration and invasion were observed in the scratch and transwell assays (Figure 4C–4F). These results indicate that both USP22 and AP4 participate in EMT and that AP4 may be a downstream molecule of USP22. To confirm these results, we down-regulated AP4 in lenti-USP22 cells (USP22-up-regulated cells) and detected the changes in USP22 and EMT marker expression. AP4 down-regulation partially reversed the increases in N-cadherin and vimentin expression levels induced by USP22 up-regulation; however, no changes in USP22 expression levels were observed (Figure 4G–4H). As shown in Figure 4I–4L, AP4 down-regulation resulted in markedly lower cell migration and invasion in affected cells than in lenti-USP22 control cells. These results suggest that EMT induction by USP22 requires functional AP4. Consistent with the above results, ChIP analysis revealed that USP22 binds the promoter region of AP4 (Figure 4M). Taken together, these results indicate that USP22 increases cell migration and invasion by inducing EMT by binding to the promoter of AP4 to activate its transcription.

### USP22 stimulates CRC cell metastasis *in vivo*

**Figure 5:**
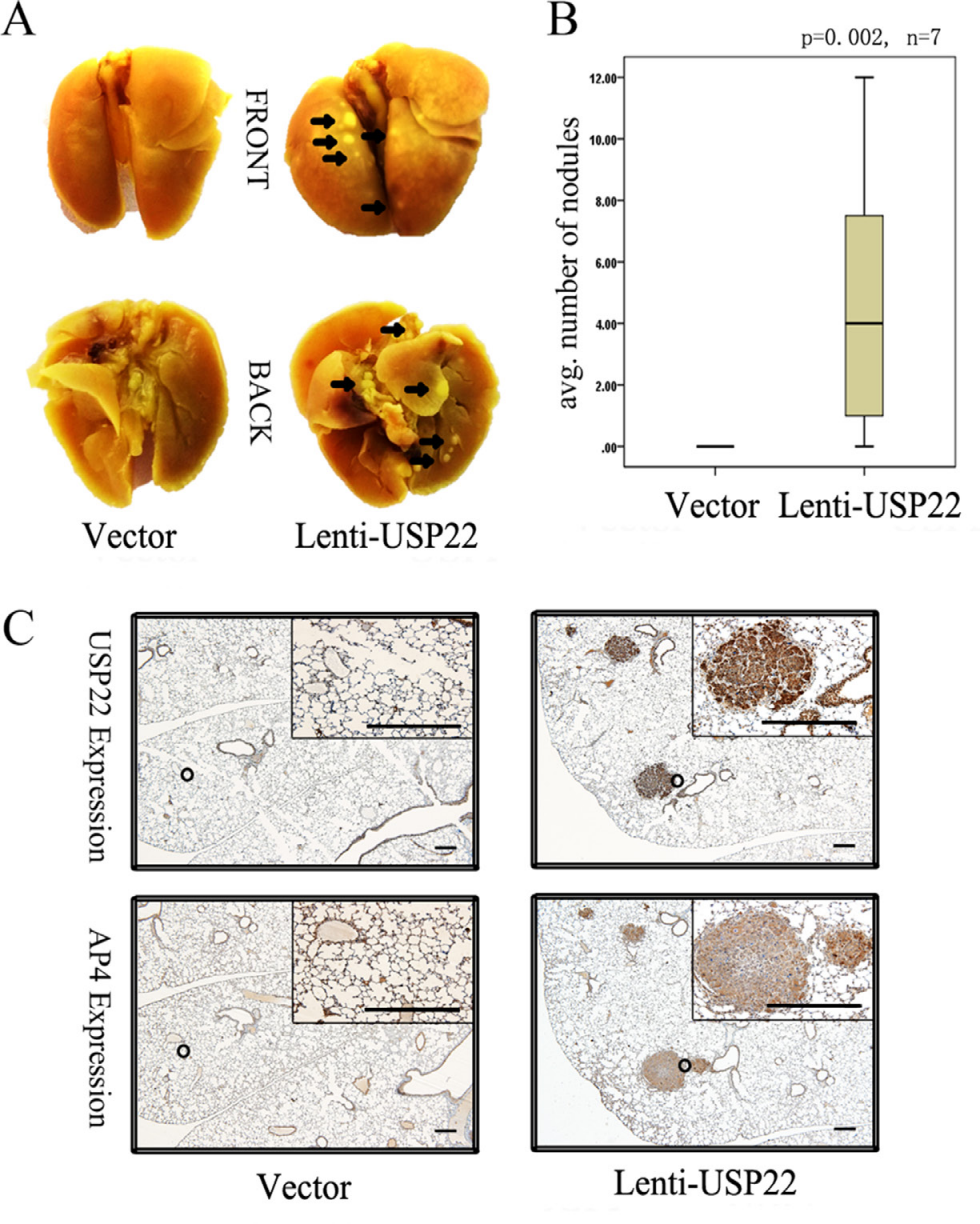
USP22 promotes lung metastasis of CRC cells in nude mice (**A**) Vector control cells and lenti-USP22 cells were injected into the tail veins of BALB/C nude mice (*n* = 7 for each cell type). The lungs were resected 4 weeks later. The arrows indicate metastatic tumor nodules (dyed light yellow). (**B**) Quantification of metastatic nodules in the lungs per mouse. (**C**) IHC analysis revealed co-expression of USP22 and AP4 in consecutive sections of lung metastasis. Bars, 100 μm.

We subsequently injected vector control and lenti-USP22 cells (Figure 3) into the tail vein of BALB/C nude mice. After 4 weeks, all lungs were resected from the nude mice. The lungs of lenti-USP22-treated mice displayed macroscopically visible metastases, whereas the lungs of control mice displayed no metastases (Figure 5A). Quantification of the results of a detailed histological examination of the lungs revealed that significantly greater numbers of metastatic nodules were present in lenti-USP22-treated mice than in control mice (Figure 5B). Furthermore, IHC analysis showed that USP22 and AP4 were co-expressed in consecutive lung metastasis sections (Figure 5C). These results demonstrate that USP22 plays a key role in metastasis formation in this mouse model.

### USP22 and AP4 overexpression is correlated with liver metastasis in CRC and poor outcomes in CRC patients

**Figure 6:**
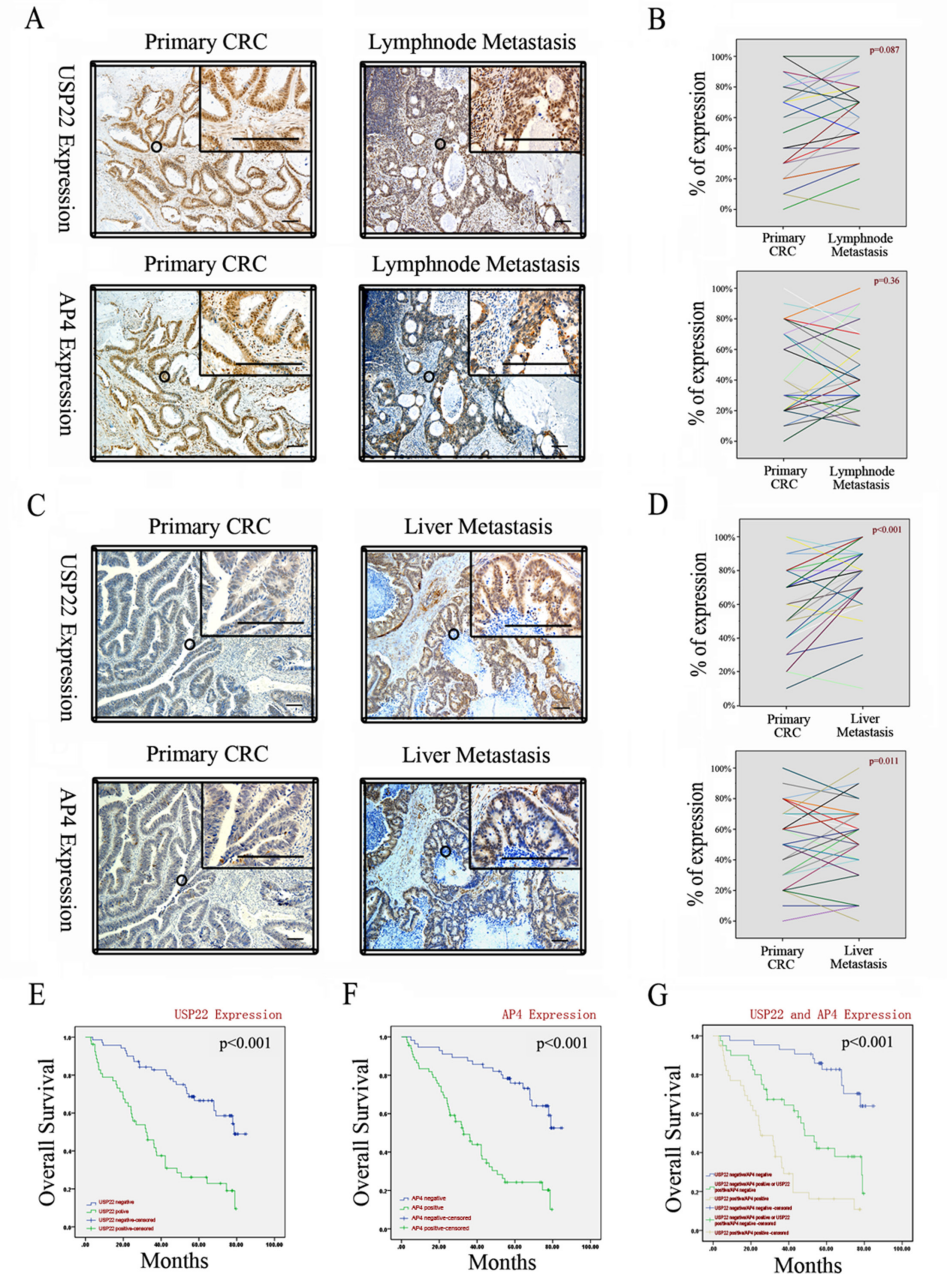
USP22 and AP4 are associated with metastasis of CRC and poor patient survival (**A**) Expressions of USP22 and AP4 in primary CRC and lymph node metastases by IHC. (**B**) Comparison of USP22 and AP4 expression in paired primary CRC and lymph node metastases. (**C**) Expressions of USP22 and AP4 in primary CRC and liver metastases. (**D**) USP22 and AP4 expression were both significantly higher in liver metastasis than in the corresponding primary CRC. (**E**) Kaplan-Meier plots of CRC specimens (*n* = 123) with negative and positive USP22 expression. (**F**) Kaplan-Meier plots of CRC specimens with negative and positive AP4 expression. (**G**) Kaplan-Meier plots of CRC specimens with negative for both USP22 and AP4; positive for one of the two proteins; positive for both USP22 and AP4, respectively. All log-rank tests indicated statistical significance (*p* < 0.001). Bars, 100 μm.

We performed IHC on 30 pairs of primary CRC and matching lymph node metastases and 38 pairs of primary CRC and matching liver metastases. Staining for USP22 and AP4 was mostly localized within colorectal epithelial cell nuclei, a finding consistent with those of previous reports [7, 19], and the two staining regions strictly overlapped (Figure 6A–6C). Compared with their expression levels in the corresponding primary CRC tissues, USP22 and AP4 expression levels were significantly increased in the liver, but not the lymph node, metastases (Figure 6B–6D). These observations suggest that both USP22 and AP4 participate in distant CRC metastasis.

Primary tumor sections from 123 CRC patients were analyzed by IHC to assess the correlations between USP22 and AP4 expression and clinical characteristics (Table 1). The IHC scoring criteria are shown in Supplementary Figure 4. Interestingly, USP22 and AP4 expression levels were concurrently highly related to primary tumor classification (pT classification: χ^2^ = 4.43, *p* = 0.035 and χ^2^ = 5.171, *p* = 0.023, respectively), liver metastasis (pM classification: χ^2^ = 16.293, *p <* 0.001 and χ^2^ = 9.031, *p* = 0.003, respectively), AJCC stage (χ^2^ = 16.791, *p <* 0.001 and χ^2^ = 11.475, *p* = 0.009, respectively), and the blood parameters carcinoembryonic antigen (CEA: χ^2^ = 4.37, *p* = 0.037 and χ^2^ = 4.332, *p* = 0.037, respectively) and carbohydrate antigen 19–9 (CA199: χ^2^ = 6.701, *p* = 0.01 and χ^2^ = 4.576, *p* = 0.032, respectively) and were also significantly associated with each other (χ^2^ = 16.561, *p <* 0.001). By contrast, no significant relationships between the expression levels of these two proteins and age, gender, location, tumor size, differentiation and lymph node metastasis were noted.

**Table 1:** Nuclei staining of USP22 and AP4 in tumor cells and associations with clinicopathologic characteristics

Variable	USP22					AP4				
	No.	Negative	Positive	χ^2^	*p* -value	No.	Negative	Positive	χ^2^	*p* -value
**Age (years)**				1.226	0.268				1.461	0.227
< 60	68	34	34			68	42	26		
≥ 60	55	22	33			55	28	27		
**Gender**				0.079	0.778				1.461	0.227
Male	73	34	39			73	43	30		
Female	50	22	28			50	27	23		
**Location**				0.079	0.778				0.291	0.590
colon	50	22	28			50	27	23		
rectum	73	34	39			73	43	30		
**Tumor size(cm)**				3.468	0.063				0.780	0.377
< 5	59	32	27			59	36	23		
≥ 5	64	24	40			64	34	30		
**Differentiation**				1.4	0.236				0.143	0.705
poor	51	20	31			51	28	23		
well	72	36	36			72	42	30		
**pT classification**				4.43	0.035*				5.171	0.023*
T1–T3	49	28	21			49	34	15		
T4	74	28	46			74	36	38		
**pN classification**				0.762	0.383				0.135	0.713
N0	65	32	33			65	38	27		
N1–N2	58	24	34			58	32	26		
**pM classification**				16.293	< 0.001***				9.031	0.003**
M0	85	49	36			85	56	29		
M1	38	7	31			38	14	24		
**AJCC stage**				16.791	< 0.001***				11.475	0.009**
I	25	13	12			25	19	6		
II	29	17	12			29	16	13		
III	31	19	12			31	21	10		
IV	38	7	31			38	14	24		
**CEA**				4.37	0.037*				4.332	0.037*
≤ 5.0 (Negative)	62	34	28			62	41	21		
> 5.0 (Positive)	61	22	39			61	29	32		
**CA199**				6.701	0.01*				4.576	0.032*
≤ 37.0 (Negative)	97	50	47			97	60	37		
> 37.0 (Positive)	26	6	20			26	10	16		
**AP4/USP22 expression**				16.561	< 0.001***				16.561	< 0.001***
Negative	70	43	27			56	43	13		
Positive	53	13	40			67	27	40		
**Total**	123	56	67			123	70	53		

Significance level as indicated: *p < 0.05; **p < 0.01; ***p < 0.001.

We further analyzed the relationships between USP22 and AP4 expression in these patients and survival, which ranged from 0.2 to 7.1 yr. The log-rank test revealed that both USP22 and AP4 were positively related to poor overall survival (OS) in CRC patients (USP22: negative 69.63 ± 3.19 months vs. positive 39.37 ± 3.28 months, *p <* 0.001; AP4: negative 66.27 ± 3.04 months vs. positive 37.37 ± 3.75 months, *p <* 0.001; respectively; Figure 6E–6F). OS was shortest in patients with double-positive (USP22 positive/AP4 positive) tumors, whereas clinical outcomes were better in patients with single-positive (USP22- or AP4-positive) or double-negative (USP22 negative/AP4 negative) tumors (double-positive 32.06 ± 3.84 months vs. single-positive 50.74 ± 4.28 months vs. double-negative 74.70 ± 2.92, *p <* 0.001; Figure 6G). Approximately 70% of patients with USP22-negative tumors and ~30% of patients with USP22-positive tumors survived 5 years after resection (Figure 6E), whereas ~80% of patients with AP4-negative tumors and ~30% of patients with AP4-positive tumors survived for the same period of time (Figure 6F). Among patients with double-positive tumors, ~80% had died at five years after resection, whereas ~40% of patients with single-positive tumors and ~90% of patients with double-negative tumors survived 5 years after resection (Figure 6G). Collectively, these results suggest that increased USP22 and AP4 expression contributes to CRC metastasis and poor outcomes in CRC patients.

## DISCUSSION

USP22 may promote tumor progression and metastasis. The underlying mechanisms by which this occurs have not been well established. Here, we present data revealing that USP22 has the ability to promote CRC metastasis via EMT induction. The key findings of the study are as follows: (i) USP22 enhances CRC cell migration and invasion by inducing EMT, (ii) USP22 directly increases AP4 transcription to induce EMT and promote CRC cell metastasis to the lungs *in vivo*, and (iii) USP22 and AP4 overexpression is related to CRC progression and liver metastasis and poor outcomes in CRC patients.

The vast majority of cancer deaths are due to metastasis, which is a very complicated process involving numerous molecules and signaling pathways. EMT is closely correlated with cancer cell invasion and metastasis [26, 27], and TGF-β1 is a key growth factor that can drive EMT [16, 28]. We observed that USP22 expression increased in EMT in CRC cells induced by TGF-β1, indicating that USP22 participates in EMT in CRC cells. Our results also demonstrated that USP22 can increase cell migration and invasion abilities via EMT induction. According to the results of previous studies, USP22 can induce EMT by regulating TGF-β1 in lung cancer cells [8], and elevated USP22 expression can promote EMT by up-regulating ZEB1 and Snail in pancreatic cancer cells though a process that involves focal adhesion kinase (FAK) signaling [29]. The present study has revealled an additional novel mechanism by which USP22 induces EMT. Our in-depth examination demonstrated that AP4 acts as a downstream molecule of USP22 and is required for the promotion of EMT by USP22 and the increased migration and invasion of CRC cells. AP4 transcription is increased by TGF-β1 [20], which, in turn, can be activated by USP22. In addition, USP22 is required for c-MYC-mediated transcription. Decreased levels of USP22 reduce the ability of c-MYC, which can stimulate activation of AP4 to directly or indirectly induce EMT [19], activating the transcription of its targets [22, 30]. We also found that USP22 binds to the promoter region of AP4 to mediate its transcription and thus induce CRC cell EMT. In summary, USP22 induces EMT by activating AP4 transcription to enhance CRC cell migration and invasion.

We subsequently revealed that USP22 up-regulation in poorly metastatic cells can promote CRC cell metastasis to the lungs in nude mouse models. Other EMT-related factors have also been reported to show similar characteristics in animal models [31, 32]. Additionally, IHC analysis of consecutive sections of lung metastases resected from the mouse model revealed that USP22 and AP4 were co-expressed in the metastases. These results lend further support to the results of our vitro experimental studies, as they showed that USP22 promotes CRC cell metastasis by activating AP4 to induce EMT.

USP22 is a biomarker for advanced malignancy in numerous tumor types, including non-small cell lung cancer [33], gastric carcinoma [34] and hepatocellular cancer [35]. We detected simultaneous overexpression of USP22 and AP4 in CRC primary carcinoma. IHC revealed that USP22 and AP4 were concurrently overexpressed in distant metastases, particularly in liver metastasis lesions, compared with corresponding primary carcinoma in CRC patients. The association between USP22 and AP4 and liver, but not lymph node, metastasis may be due to these proteins driving blood stream, but not lymphatic, metastasis of CRC cells. Consistent with these results, injection of USP22-expressing cells via the tail vein in nude mice promoted CRC cell metastasis to the lungs. Additionally, assessment of CRC clinicopathological characteristics demonstrated that USP22 and AP4 expression levels were significantly associated with pT classification, pM classification, AJCC stage and tumor markers used as prognostic indicators of postoperative recurrence and metastasis in CRC, including CEA and CA199. Furthermore, Kaplan-Meier analysis demonstrated that overexpression of these proteins contributes to poor CRC patient survival. These results demonstrate that USP22 and AP4 may promote tumor progression and metastasis and imply that their expression leads to poor outcomes in CRC.

In summary, we have presented evidence that USP22, an up-stream molecule of AP4, exhibits strong potential to promote CRC metastasis, particularly CRC migration and invasion capacities, both *in vitro* and *in vivo*, by inducing EMT by activating AP4. Moreover, USP22 and AP4 overexpression may stimulate tumor metastasis and adversely affect OS in CRC patients. Accordingly, USP22 and AP4 may serve as prognostic markers for predicting the risk of developing distant metastases in CRC and therapeutic targets for the prevention and treatment of CRC patients with metastases. However, the functional role of USP22 as a DUB may also be a critical contributor to the process of USP22-induced EMT via epigenetic regulation, emphasizing the need to explore the epigenetic functions of USP22 in CRC metastasis.

## MATERIALS AND METHODS

### Tissues and patients

Paired non-cancerous mucosal samples and primary tumor samples were collected from 19 patients undergoing resections performed by one surgeon. All cases were pathologically confirmed. Additionally, formalin-fixed, paraffin-embedded specimens, including primary carcinoma specimens (*n* = 123), matching lymph node metastasis specimens (*n* = 30) and liver metastasis specimens (*n* = 38), used for IHC were collected from 123 CRC patients who underwent surgery from 2008–2010. Primary carcinomas were assessed according to the 7th edition of the American Joint Committee on Cancer (AJCC) staging system. All CRC patient data and tissue samples were collected from the Affiliated Tumor Hospital of Harbin Medical University. No patients received preoperative radiotherapy or chemotherapy. The study was approved by the Ethics Committee of the Affiliated Tumor Hospital of Harbin Medical University, Harbin, China.

### Cell lines and EMT induction

The human CRC cell lines SW174, SW480, HCT116 and SW1116 were obtained from the Cancer Research Institute of Harbin Medical University. The cell lines were maintained in RPMI-1640 (Gibco) supplemented with 10% fetal bovine serum (ExCell Bio, Shanghai, China), 100 μg/ml streptomycin and 100 units/ml penicillin and cultured at 37°C in a humidified atmosphere containing 5% CO_2_ and 95% air.

To induce EMT, the SW1116 cells were cultured in serum-free medium (containing 1% FBS) for 24 h before treatment with recombinant TGF-β1 (10 ng/ml; B&D Systems Inc., USA) for 24 h, 48 h or 72 h.

### Knockdown and overexpression

siRNAs against USP22 and AP4 and a control siRNA were purchased from Invitrogen. The target sequences are shown in Supplementary Table 2. Cells were seeded in 6-well plates (at 40 to 50% confluence) and starved for 4 h. siRNA transfection was performed with X-treme GENE transfection reagent (Roche), according to the manufacturer's instructions. The CRC cells in each group were transfected with siRNAs for at least 48 h for the purpose of further research. We performed RT-PCR to examine the knockdown efficiency of the USP22-specific siRNAs (USP22#1 siRNA: 45.37%; USP22#2 siRNA: 55.30%; Figure 3G) and AP4-specific siRNAs (AP4#1 siRNA, 76.31%; AP4#2 siRNA, 71.41%; Supplementary Figure 3) and selected the most efficient siRNAs for further study (USP22#2 and AP4#1).

A lentivirus expressing USP22 (on vector pLenti6.3_MCS_IRES2-EGFP) and a scrambled vector were obtained from Invitrogen. SW1116 cells were infected with the lentiviral particles expressing USP22 and the scrambled vector in the presence of 8 μg/ml polybrene (Sigma-Aldrich, St. Louis, MO), according to the manufacturer's instructions. The cells in the empty-control group received polybrene only. The USP22-up-regulated cells were screened using 10 μg/ml blasticidin (Invitrogen) for 3–4 weeks, and the transduction efficiency of the lenti-USP22 cells was measured using a FACS Calibur flow cytometer (Becton Dickinson, MA, USA). The transfection rate was 95.3%, as shown in Supplementary Figure 2.

### Reverse transcription and real-time polymerase chain reaction (RT-PCR)

Total RNA was extracted using TRIzol (TaKaRa, Japan), and reverse transcription (RT) for gene expression was performed using a Transcriptor First Strand cDNA Synthesis Kit (Roche, 2 μg total RNA), according to the manufacturer's instructions. To detect gene expression, we conducted RT-PCR using FastStart Universal SYBR Green Master (Roche), according to the manufacturer's protocol. Supplementary Table 1 lists the primers used for analysis. RT-PCR was performed in triplicate using an Applied Biosystems^®^ 7500 fast RT-PCR system, and the 2^^–ΔΔCT^ method was used to determine relative gene expression levels.

### Western blotting (WB)

All of the tissues and cells were collected using RIPA reagent, in accordance with approved guidelines. WB analysis was conducted with 30 μg of total proteins and antibodies against USP22 (1:500, Abcam), AP4 (1:400, Abcam), N-Cadherin (1:1000, BD Biosciences), vimentin (1:500, BD Biosciences), E-cadherin (1:500, BD Biosciences) and β-actin (1:1000, Zhongshanjinqiao, Beijing, China). The immunoreactive bands were visualized with an ECL western blotting detection system (Tanon, Shanghai, China).

### Immunofluorescent staining

After the appropriate treatment, the cells were fixed with 4% buffered paraformaldehyde in PBS. Sample penetration was performed by incubating the samples in blocking solution (1% BSA and 0.1% Triton-X in PBS) for 2 h. The samples were subsequently blocked with goat serum and incubated with USP22 (1:150, Abcam), AP4 (1:100, Abcam), N-Cadherin (1:150, BD) and E-cadherin (1:100, BD) antibodies overnight at 4°C. Then, the cells were incubated with a fluorescently labeled secondary antibody for 1 h before being washed three times in PBS. Finally, the cell nuclei were stained with 4′,6-diamidino-2-phenylin-dole (DAPI) and observed under a fluorescence microscope (Olympus, Japan).

### Immunohistochemistry (IHC)

USP22 and AP4 expression in paraffin sections was analyzed by IHC, as previously described [7]. The sections were incubated with a USP22 antibody (1:200, Abcam) and AP4 antibody (1:50, Abcam). The primary antibody was omitted for the cells in the negative-control group. The expression levels of both proteins were determined semiquantitatively by assessing the percentages of positively stained immunoreactive cells and evaluating cell staining intensity [7, 8]. USP22 and AP4 IHC staining was scored as shown in Supplementary Figure 4. Overall scores between – and + and between ++ and +++ were considered negative and positive, respectively. All samples were reviewed by two independent, experienced pathologists in a blinded fashion.

### Scratch assay

Wound-healing assay was performed to detect cell migration. Prepared cells were seeded in 6-well plates. After the cells had reached 90–100 % confluence, a 10-μl sterile micropipette tip was used to create a void of cells by scratching. The plates were then washed to remove dislodged cells. The remaining CRC cells were cultured in medium containing 5% FBS. The initial gap length (0 hours) and residual gap length at 48 hours after wounding were calculated from photomicrographs.

### Matrigel invasion assays

Cell invasion assays were performed in 24-well transwell plates (8-μm pore size, Corning Costar, USA), as described elsewhere [8]. The chamber inserts were coated with a Matrigel membrane (BD Biosciences, USA). RPMI-1640 containing 20% fetal bovine serum in the lower chamber served as the chemoattractant. The cells (5 × 10^5^/ well) were incubated at 37°C for 48 h.

### Chromatin immunoprecipitation (ChIP) analysis

ChIP assays were performed using a SimpleChIP Enzymatic Chromatin IP Kit (Cell Signaling), according to the manufacturer's protocol, after the cells were cross-linked in 1% formaldehyde. A rabbit polyclonal antibody against USP22 (Abcam) was used to link USP22 with the promoter region of AP4. The extracted DNA was then amplified by PCR using the qPCR primers (primers from the promoter region and the exon region of AP4 gene) shown in Supplementary Table 1. And the result of additional control (the exon region of AP4 gene) was showed in Supplementary Figure 5.

### Metastasis formation in a xenograft nude mice model

Female BALB/C nude mice (3 to 4 weeks of age, Vital River Laboratory Animal Technology, Beijing, China) were used for lung metastasis assays after tail vein injection. All animals were bred and housed under standard pathogen-free conditions and handled in accordance with institutional guidelines under approved protocols. The mice were injected in the lateral tail vein with 0.2 ml of a suspension of 4 × 10^6^/0.2 ml vector and lenti-USP22 cells. After 4 weeks, whole lungs were resected from the nude mice, fixed with Bouin's fluid for 24 h, and stained, as shown in Figure 5A. The number of metastases was determined microscopically.

### Statistical analysis

Statistical analysis was performed using IBM SPSS statistics 19.0. All experiments were conducted at least 3 times, and the data are reported as the mean ± SD. A chi-square test and Student's *t-test* were used for comparisons between groups. Survival curves were generated using the Kaplan–Meier method and log-rank test.

## SUPPLEMENTARY MATERIALS FIGURES AND TABLES


