# Supplementary material for: USP22 drives colorectal cancer invasion and metastasis via epithelial-mesenchymal transition by activating AP4
Source: Oncotarget. 2017 Mar 6;8(20):32683–95. doi: 10.18632/oncotarget.15950 (PMC5464819; doi:10.18632/oncotarget.15950)
Supplement: Supplementary file 1 [file oncotarget-08-32683-s001.pdf]

USP22 drives colorectal cancer invasion and metastasis via epithelial-mesenchymal transition by activating AP4

Supplementary Materials

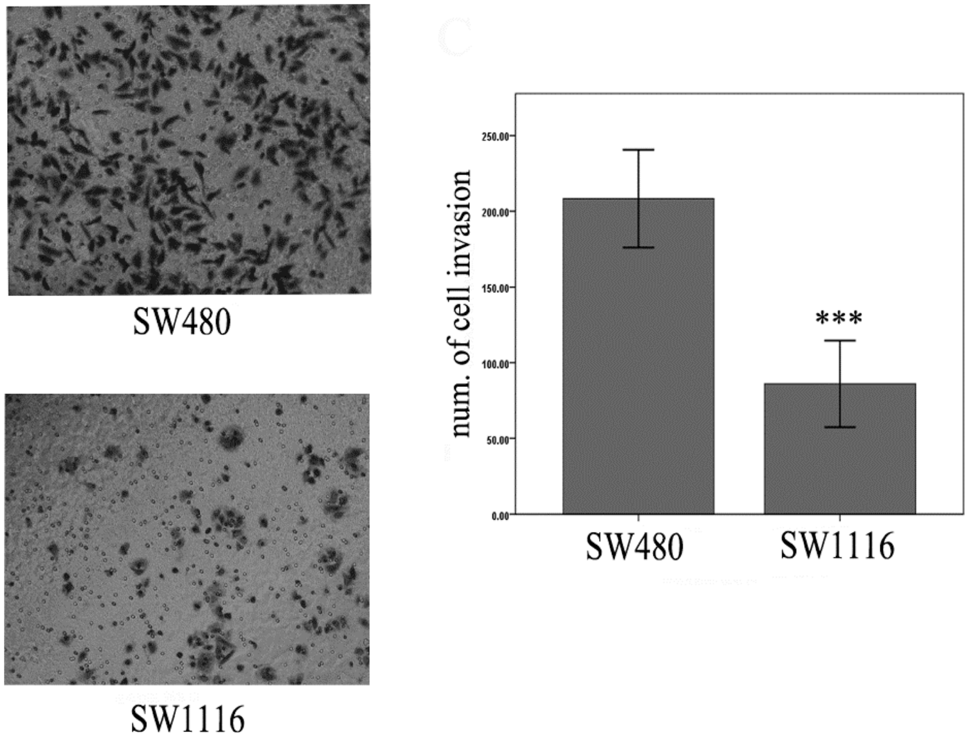

Supplementary Figure 1: Transwell assay of SW480 cells and SW1116 cells. The invasion ability of SW480 is far more stronger than SW1116.

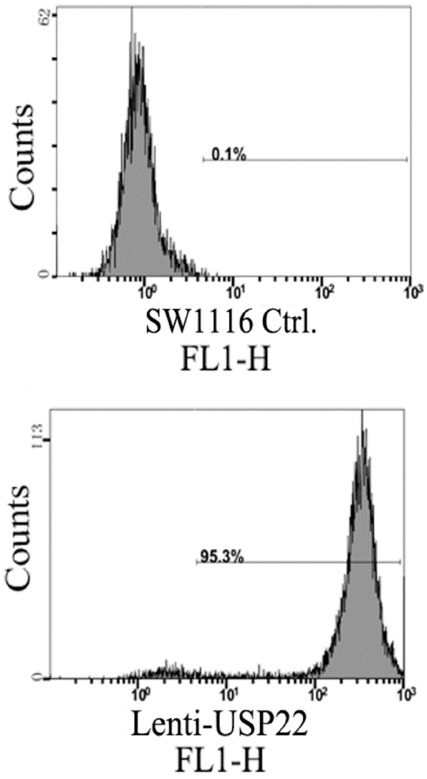

Supplementary Figure 2: Flow cytometry analysis showed the transfection rate of lenti-USP22 cells is 95.3%.

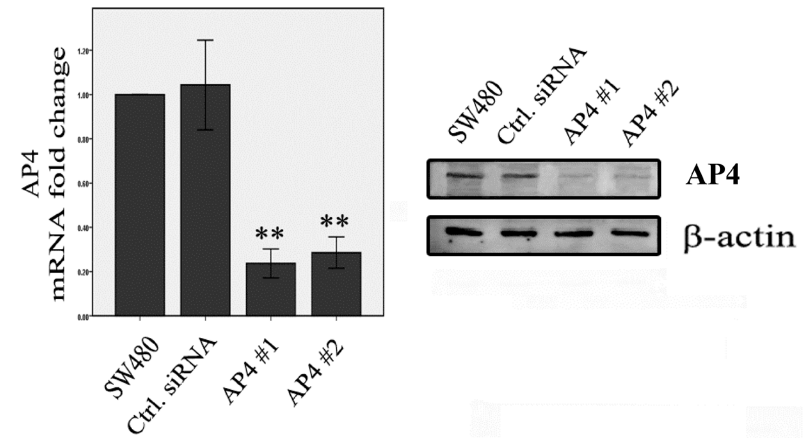

Supplementary Figure 3: Down-regulation of AP4 in SW480 cells. RT-qPCR and WB analysis revealed the knock-down efficiency of AP4.

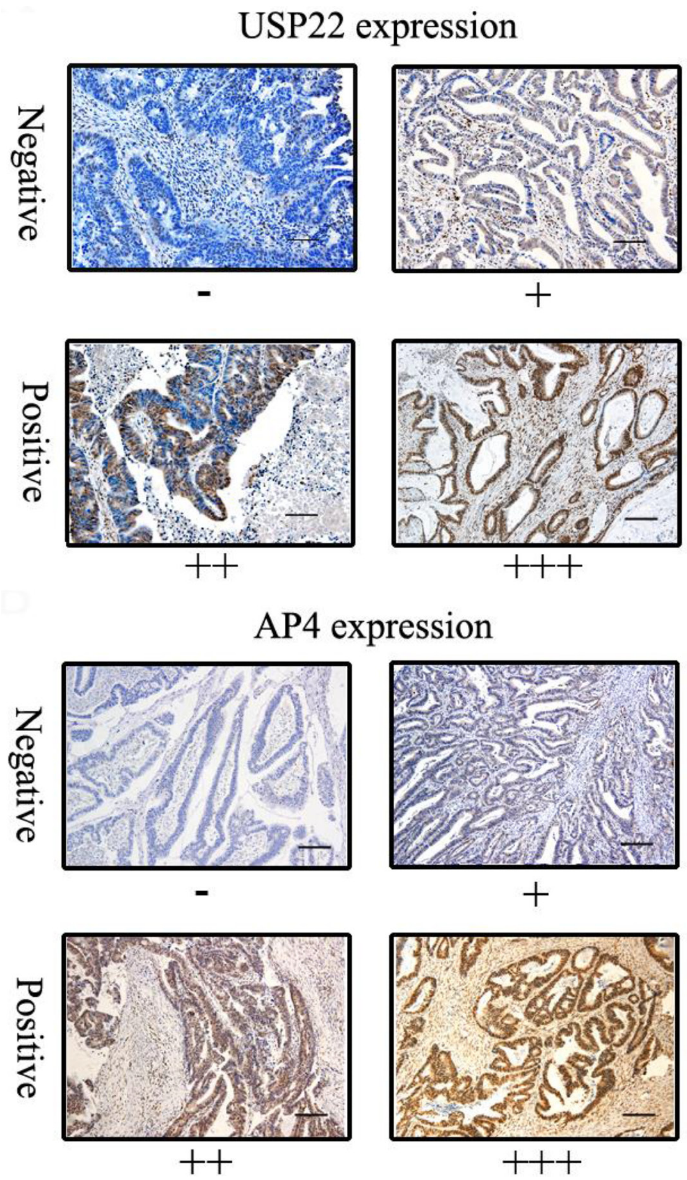

Supplementary Figure 4: Score criterias for IHC analysis of USP22 and AP4.

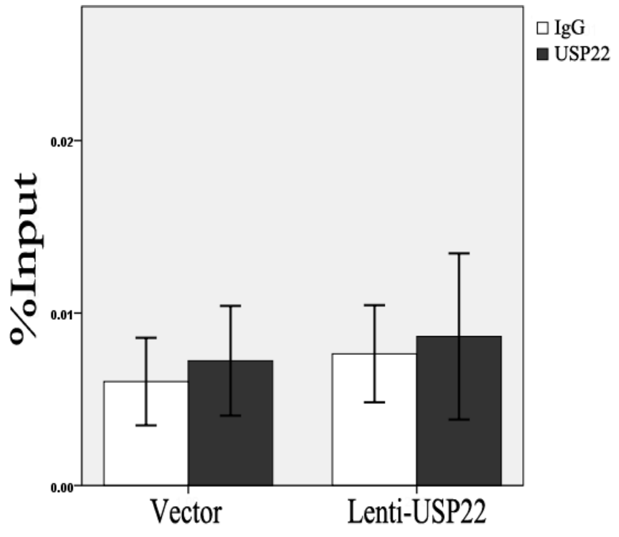

Supplementary Figure 5: The additional control for chip-analysis revealed that there's no binding between USP22 and the exon region of AP4 (IgG served as a negative control).

Supplementary Table 1: Gene primer pairs used for PCR

Gene Name	Direction	Sequence
USP22	Forward	*CCCCTCACATCCCGTATA*
	Reverse	*TTCCCATTGTCATCACCTT*
AP4	Forward	*AACAGGGGCAGAGTGAGTTG*
	Reverse	*GGGAAGTGCCTTCGACAGTG*
N-cadherin	Forward	*TGAAACGGCGGGATAAAGAG*
	Reverse	*GGCTCCACAGTATCTGGTTG*
E-cadherin	Forward	*TAGAGGGTCACCGCGTCTATG*
	Reverse	*GGGTGCGTGGCTGCAGCCAGG*
Vimentin	Forward	*GAGAACTTTGCCGTTGAAGC*
	Reverse	*GCTTCCTGTAGGTGGCAATC*
Beta-actin	Forward	*TTCCTGGGCATGGAGTCCT*
	Reverse	*AGGAGGAGCAATGATCTTGATC*
AP4-chip (promter region)	Forward	*TTGTCCTCCAGCCTCCTACG*
	Reverse	*GCTCCCTACAGTGCCTTATGC*
AP4-chip (exon region for Ctrl.)	Forward	*CCATCGCTTGTCCCAAATGC*
	Reverse	*TGAGCGATCAAGTTGGGCTT*

Supplementary Table 2: siRNA used for downregulation

Gene Name	Direction	Sequence
USP22 #1	Sense	*CAGCAGCCCACGGACAGUCUCAACA*
	Anti-Sense	*UGUUGAGACUGUCCGUGGGCUGCUG*
USP22 #2	Sense	*GCUGUUUCACAAAGAAGCAUAUUCA*
	Anti-Sense	*UGAAUAUGCUUCUUUGUGAAACAGC*
AP4 #1	Sense	*GUGAUAGGAGGGCUCUGUAG*
	Anti-Sense	*CUACAGAGCCCUCCUAUCAC*
AP4 #2	Sense	*UGGGAUUGUCAGCCUUCAATT*
	Anti-Sense	*UUGAAGGCUGACAAUCCCAGG*
Negative Control	Sense	*UUCUCCGAACGUGUCACGUTT*
	Anti-Sense	*ACGUGACACGUUCGGAGAATT*
